# The Effects of COVID-19 on Clinical Outcomes of Non-COVID-19 Patients Hospitalized for Upper Gastrointestinal Bleeding during the Pandemic

**DOI:** 10.3390/jcm11092461

**Published:** 2022-04-27

**Authors:** Nonthalee Pausawasdi, Ekawat Manomaiwong, Uayporn Kaosombatwattana, Khemajira Karaketklang, Phunchai Charatcharoenwitthaya

**Affiliations:** 1 Siriraj GI Endoscopy Center, Faculty of Medicine Siriraj Hospital, Mahidol University, Bangkok 10700, Thailand; nonthaleep7@gmail.com (N.P.); koigi214@gmail.com (U.K.); 2 Division of Gastroenterology, Department of Medicine, Faculty of Medicine Siriraj Hospital, Mahidol University, Bangkok 10700, Thailand; e_manomaiwong@hotmail.com (E.M.); oy.kemajira@gmail.com (K.K.)

**Keywords:** upper gastrointestinal bleeding, COVID-19, pandemic, endoscopy, outcomes

## Abstract

This study aims to investigate the effects of COVID-19 on clinical outcomes of non-COVID-19 patients hospitalized for upper gastrointestinal bleeding (UGIB) during the pandemic. A retrospective review is conducted. We recruited patients with UGIB admitted during the pandemic’s first wave (April 2020 to June 2020), and the year before the pandemic. The outcomes between the two groups were compared using propensity score matching (PSM). In total, 60 patients (pandemic group) and 460 patients (prepandemic group) are included. Patients admitted during the pandemic (mean age of 67 ± 14 years) had a mean Glasgow–Blatchford score of 10.8 ± 3.9. They were older (*p* = 0.045) with more underlying malignancies (*p* = 0.028), had less history of NSAID use (*p* = 0.010), had a lower platelet count (*p* = 0.007), and had lower serum albumin levels (*p* = 0.047) compared to those admitted before the pandemic. Esophagogastroduodenoscopy (EGD) was performed less frequently during the pandemic (43.3% vs. 95.4%, *p* < 0.001). Furthermore, the procedure was less likely to be performed within 24 h after admission (*p* < 0.001). After PSM, admissions during the pandemic were significantly associated with decreased chances of receiving an endoscopy (adjusted odds Ratio (OR), 0.02; 95% CI, 0.003–0.06, *p* < 0.001) and longer hospital stay (adjusted OR, 2.17; 95% CI, 1.13–3.20, *p* < 0.001). Additionally, there was a slight increase in 30-day mortality without statistical significance (adjusted OR, 1.92; 95% CI, 0.71–5.19, *p* = 0.199) and a marginally higher rebleeding rate (adjusted OR, 1.34; 95% CI, 0.44–4.03, *p* = 0.605). During the pandemic, the number of EGDs performed in non-COVID-19 patients with UGIB decreased with a subsequent prolonged hospitalization and potentially increased 30-day mortality and rebleeding rate.

## 1. Introduction 

Coronavirus disease 2019 (COVID-19), caused by severe acute respiratory syndrome coronavirus 2 (SARS-CoV-2), has significantly impacted public health worldwide. The COVID-19 pandemic has had a disruptive effect on the workflow and safety of healthcare personnel and patients [1]. An endoscopy is considered a high-risk procedure for COVID-19 transmission due to the aerosol-generating nature of the technique despite no evidence of SARS-CoV-2 transmission by endoscopy [2,3,4]. Operational reorganization has been undertaken to limit viral spreading since the pandemic started. Nonemergent procedures can be postponed during the pandemic; however, the evaluation of gastrointestinal (GI) bleeding is often urgent and cannot be deferred. International guidelines recommend an early endoscopy within 24 h of clinical stabilization as the first-line diagnostic and therapeutic modality for upper GI bleeding (UGIB) [5,6,7]. 

Several international GI societies have issued recommendations for performing an endoscopy in the COVID-19 era to reduce transmission risk in resource-limited settings, lacking personal protective equipment (PPE), infrastructures, and staff [8,9,10,11]. Endoscopic procedures, mostly therapeutic interventions, should be reserved for patients with urgent or life-threatening conditions [12]. However, the need for PPE, COVID-19 testing, a negative pressure room, the particular method for room disinfection, and the personnel shortage during the initial COVID-19 outbreak all may have caused deferred endoscopies. Delays in identifying the cause of UGIB and performing endoscopic interventions may result in poor outcomes and possibly decreased overall survival. This study aims to evaluate the impact of the COVID-19 pandemic on clinical outcomes of non-COVID-19 patients hospitalized with acute UGIB.

## 2. Materials and Methods

### 2.1. Patient Population

This retrospective study was conducted at Siriraj Hospital, a large referral center serving the Bangkok metropolitan area and surrounding communities. The study conformed to the ethical guidelines of the Helsinki Declaration and was approved by the Institutional review board. The COVID-19 pandemic cohort comprised all consecutive non-COVID-19 persons aged ≥ 18 years who were hospitalized for the treatment of acute UGIB from 1 April 2020 to 30 June 2020. The pre-COVID-19 pandemic cohort included patients hospitalized with acute UGIB in the year preceding 1 April 2020. Patients who developed UGIB during their hospitalization for other indications were excluded.

### 2.2. Management of Upper Gastrointestinal Bleeding 

Initial resuscitation and risk stratification using Glasgow–Blatchford score (GBS) were performed in all patients. All patients were given nothing by mouth. Intravenous fluid administration and packed red blood cells transfusions were administered as indicated. Patients with clinical suspicion of nonvariceal bleeding received intravenous proton pump inhibitor (PPI), whereas those with suspected variceal bleeding were given intravenous somatostatin or its long-acting analogues prior to endoscopic assessment. Patients with GBS > 1 were evaluated with esophagogastroduodenoscopy (EGD) within 24–72 h unless contraindicated otherwise, and endoscopic hemostasis was applied as indicated. In patients who required endoscopic intervention for peptic ulcer disease with stigmata of active or recent bleeding, high-dose PPIs were administered through infusion for 72 h following the procedure. Band ligation and cyanoacrylate injection were used to treat bleeding esophageal and gastric varices, respectively, in addition to vasoactive medications and intravenous antibiotics. The intravenous somatostatin or its long-acting analogues was continued for 2–5 days after endoscopic treatment.

During the COVID-19 pandemic, aerosol-generating procedures were restricted because of the following: (1) the risk of spreading COVID-19, (2) the limited availability of PPE, (3) the need for preprocedural COVID-19 testing with Reverse Transcription-Polymerase Chain Reaction (RT-PCR), and (4) negative-pressure rooms. Therefore, the timing of EGDs could have deviated from the routine protocol, but the pre-endoscopic management was carried out as usual. EGD was promptly performed if patients had either one or more of the following: hemodynamic instability, ongoing or recurrent GI bleeding after previous hemodynamic stability, or suspected variceal hemorrhage. In cases of delayed or postponed endoscopic evaluation, additional treatments such as intravenous PPI, somatostatin, or its long-acting analogues, were administered until endoscopic evaluation was performed, or bleeding ceased.

### 2.3. Clinical, Laboratory, and Endoscopic Data

Patient demographics, clinical manifestations, concomitant disorders, laboratory tests, drugs administered during hospitalization, and endoscopic findings were extracted from electronic medical records. The 30-day mortality, the need for endoscopy, rebleeding, the amount of blood transfusion, and the length of hospital stay were reviewed. Rebleeding was defined as the reoccurrence of hematemesis or melena with signs of hemodynamic instability or decrease in hemoglobin level >2 g/dL in a previously stable case. The presence of hemodynamic instability was defined as systolic blood pressure <100 mmHg with heart rate > 100 beats/min or orthostatic changes with a >10% decrease in systolic blood pressure and a >10% increase in heart rate between supine and seated positions. The primary outcome was 30-day mortality, while secondary outcomes included endoscopic performance, transfusions during hospitalization, and length of stay. 

### 2.4. Statistical Analysis

Continuous variables were described as mean ± standard deviation (SD) or median (interquartile range (IQR)) and were analyzed using the Student’s *t*-test. Categorical variables were reported as numbers (percentage) and were analyzed using the χ^2^ or Fisher exact tests. Multivariate logistic and linear regression models were used to estimate adjusted odds ratios (OR) or differences of means for the study outcomes. Baseline variables with a standardized difference in absolute values greater than 0.15 were considered for multivariate analysis. In the primary analysis, we examined whether admissions during the pandemic had a different risk of 30-day mortality, endoscopy, blood transfusions, and length of stay compared to admissions before the pandemic. 

As a secondary analysis, study outcomes were compared in propensity score (PS)-matched patients. Propensity scores were estimated using a logistic regression model for the COVID-19 versus the pre-COVID-19 pandemic cohorts that included demographic characteristics (age, sex), comorbidities (cirrhosis, chronic kidney disease, cardiovascular disease, cerebrovascular accident, malignancy, Charlson comorbidity index), bleeding severity (GBS), use of nonsteroidal anti-inflammatory drugs (NSAIDs), and laboratory tests (albumin, platelet count). Patients hospitalized during the COVID-19 pandemic were matched one-to-three with a caliper of 0.15 to UGIB patients who were managed before the pandemic using PS matching. Statistical analyses were performed using STATA version 14.0 (StataCorp LP, College Station, TX, USA). A two-sided *p*-value of below 0.05 was considered statistically significant.

## 3. Results

### 3.1. Characteristics of the Population

A total of 520 patients with UGIB were recruited, out of which 60 patients were admitted during the first wave of the COVID-19 pandemic in Thailand, while the remaining 460 patients were admitted a year before the pandemic. All patients admitted during the pandemic tested negative for SARS-CoV-2 infection. Clinical characteristics and laboratory data of the two cohorts are shown in Table 1. Patients admitted with acute UGIB during the pandemic (mean age of 67 ± 14 years, 61.7% male) had a mean GBS of 10.8 ± 3.9. Patients admitted during the pandemic were older (*p* = 0.045) and had a higher Charlson comorbidity index (*p* = 0.039), especially coexisting with solid organ malignancies (*p* = 0.028), less history of NSAID use (*p* = 0.01), lower serum albumin levels (*p* = 0.047) and platelet count (*p* = 0.007) compared to those hospitalized before the pandemic. Otherwise, there were no differences in terms of sex, underlying liver cirrhosis, chronic kidney disease, atherosclerotic disease, use of antiplatelet or anticoagulants, clinical manifestations, and GBS between both groups. 

Table 2 shows the baseline characteristics of the matched populations after the PS matching. In total, 46 patients admitted during the pandemic were matched with 138 patients admitted before the pandemic. The standardized difference for each characteristic of patients admitted during and before the pandemic was comparable.

### 3.2. Primary Outcomes

Overall, 11 patients admitted during the pandemic died, but only one (1.7%) death was associated with bleeding. Of these, six patients died from their underlying malignancies. During the prepandemic period, 41 deaths were reported, and 12 (2.6%) of them were related to bleeding. The causes of deaths are shown in Table 3. 

Treatment outcomes of the overall and matched populations are shown in Table 4. In the univariate analysis, the admissions during the pandemic were associated with a higher 30-day mortality than those before the pandemic (unadjusted OR, 2.29; 95%CI, 1.11–4.75, *p* = 0.025). After adjusting for demographics, comorbidities, bleeding severity, and antiplatelet/anticoagulant usage, an increase in the 30-day mortality was still observed during the pandemic, but it did not reach statistical significance (adjusted OR, 1.33; 95% CI, 0.52–3.39, *p* = 0.550). Once the patient population was matched using PS, eight patients (17.4%) died within 30 days of hospitalization during the pandemic, compared to sixteen cases (11.6%) admitted before the pandemic (*p* = 0.315). Despite the increased odds of 30-day mortality among the admissions during the COVID-19 outbreak, the difference was not statistically significant in the multivariable analysis when adjusting for potential residual confounders (adjusted OR, 1.92; 95% CI, 0.71–5.19, *p* = 0.199).

### 3.3. Secondary Outcomes 

Table 5 shows the number of the overall population requiring in-hospital interventions according to the GBS. During the pandemic, none of the patients with a GBS of ≤3 underwent EGD, received blood transfusion, or died within 30 days. EGD was performed in 26 patients (43.3%) who had hemodynamic instability (*n* = 2), rebleeding after medical treatment (*n* = 8), or a suspicion of variceal bleeding (*n* = 16). In contrast, 439 patients (95.4%) admitted before the pandemic underwent EGD (adjusted OR, 0.01; 95% CI, 0.003–0.03, *p* < 0.001). The median duration from presentation to EGD (70 h, IQR 48–111) during the pandemic was much longer than in the pre-COVID-19 era (25 h, IQR 16–48). EGD was performed within 24 h in only four patients (6.7%) during the pandemic, compared to 208 (45.2%) patients admitted before the pandemic (adjusted OR, 0.06; 95% CI, 0.02–0.22, *p* < 0.001). The distribution of the types of lesions observed among patients undergoing EGD was comparable across patients admitted during and before the pandemic (Table 6). Peptic ulcer was the most common identified lesion, followed by varices. After adjusting for confounders, increased OR was observed for blood transfusion (adjusted OR, 1.18; 95% CI, 0.60–1.75, *p* = 0.051) and length of stay (adjusted OR, 2.21; 95% CI, 0.11–4.31, *p* < 0.198), but the differences did not reach statistical significance. There was no significant difference in the rebleeding rate (adjusted OR, 1.16; 95% CI, 0.44–3.03, *p* = 0.764).

After PS matching, patients having undergone endoscopies during the pandemic remained considerably lower than those admitted before the pandemic (adjusted OR, 0.02; 95% CI, 0.003–0.06, *p* < 0.001). However, endoscopic findings of peptic ulcer disease, varices, and other lesions were observed similarly. Additionally, admissions during the pandemic had a longer length of stay (adjusted OR, 2.17; 95% CI, 1.13–3.20, *p* < 0.001). The odds of rebleeding were slightly increased during the pandemic but not statistically significant (adjusted OR, 1.34; 95% CI, 0.44–4.03, *p* = 0.605) compared to prepandemic. The units of blood transfusion were similar between the two periods.

## 4. Discussion

This study exhibited the impact of the COVID-19 pandemic in the management and treatment outcomes of non-COVID-19 patients presenting with UGIB. The results demonstrated that patients admitted during the pandemic were older with more underlying malignancies, had more history of NSAID use, and had more concerning laboratory results. They were less likely to undergo EGD; furthermore, only 6.7% had EGD performed within 24 h. Overall, we found an increased 30-day mortality, blood transfusion, and length of stay. However, the impact of the pandemic on mortality and blood transfusion became insignificant after adjusting for confounding factors and PS matching. Nonetheless, prolonged hospitalization remained associated with admissions during the pandemic after PS matching. The difference in the rebleeding rate was not statistically significant between the two periods. 

Patients with UGIB admitted during the pandemic were sicker with more abnormal laboratory results. These observations may be indicative of the patients’ unwillingness to present to the hospital during the pandemic unless they had serious underlying diseases with severe symptoms or a higher threshold of hospital admission. Similar findings were observed in a study conducted in the United States [13], underscoring the global impact of COVID-19 on patients’ concerns about hospital visits and admission criteria during the pandemic. 

Generally, GBS has been recommended by international guidelines for risk stratification for patients presenting with UGIB [14,15]. Patients with GBS ≤ 1 are considered low-risk and can be managed as outpatients, without the necessity for an in-hospital endoscopy. Due to the strain on the healthcare system, and a lack of PPE during the pandemic, new extended low-risk GBS thresholds were proposed and clinical outcomes were assessed. The data from a large international multicenter study involving 3012 consecutive patients with UGIB showed that using GBS ≤ 3 as the threshold to avoid hospitalization resulted in avoidance of admission and an inpatient endoscopy in 32% of patients [16]. In low-risk individuals, the percentage of patients requiring endoscopic treatment (4.1%) and dying within 30 days (1.7%) might be an acceptable number in countries at risk for healthcare system collapse from COVID-19. In our study’s population, only three patients (5%) admitted during the pandemic had GBS ≤ 3, suggesting the limited use of this proposed threshold for identifying low-risk patients. 

The study by Ilagan-Ying et al. showed that patients admitted during the first wave of the pandemic (1 March–31 May 2020), who required inpatient endoscopic procedures, were sicker with higher ICU admissions and had higher 30-day mortality rates. The indications for endoscopy included volvulus, obstruction, foreign body, food impaction, biliary tract obstruction, acute cholangitis, and GI bleeding. The diagnosis of COVID-19, an age over 65, and ICU admissions were shown to be associated with increased mortality for admissions during the pandemic [17]. However, Kim et al. reported that patients with GI bleeding admitted during the pandemic were more likely to have concerning laboratory results, received blood transfusions, and had a prolonged hospital stay, but the inpatient mortality rate was comparable to those admitted before the pandemic [13]. Our study found an increased 30-day mortality in patients admitted during the pandemic. However, the difference in the mortality rate was not statistically significant between the two periods after PS matching and adjusting for potential confounders, suggesting that patients’ coexisting conditions may have had an impact on mortality rather than the GI bleeding itself.

The results also showed that patients admitted during the pandemic tended to have a higher number of blood transfusions; however, the effect was less significant after adjusting for confounders and PS matching. These findings may imply that the number of blood transfusions among patients admitted during the COVID-19 outbreak might be attributable to comorbidities rather than bleeding severity. Furthermore, the volume of EGD in UGIB was less, and there were more delayed endoscopies during the pandemic compared to the year before in the overall and matched population. In addition, the length of hospital stay increased significantly for admissions during COVID-19. This may reflect the physicians’ concern for early rebleeding or delayed adverse events. 

This study had some limitations. First, the retrospective design of this study had several drawbacks, which raised the possibility of selection bias. Herein, we employed PS matching to overcome the effects of potential confounding factors. Second, this was a single-center study with small numbers. Thus, multicenter prospective research with a larger sample size is required to extend the findings to other populations. Third, the pre-COVID-19 cohort included more patients with stigmata of recent bleeding, raising the possibility of bias. This finding might be attributed to the influence of intensive PPI regimen usage on the resolution of peptic ulcer disease, resulting in a low incidence of high-risk stigmata on the endoscopic assessment during the pandemic. Finally, the assessment of rebleeding can be challenging, because only 43% of the patients admitted during the pandemic underwent EGD.

## 5. Conclusions

This study suggested that the disruptive effects on the healthcare system during the first wave of the COVID-19 outbreak led to the restriction of endoscopy services, including the performance of urgent procedures. As a result, clinical outcomes of non-COVID-19 patients who required the endoscopic management of UGIB were compromised. The number of performed EGDs decreased, whereas the length of hospitalization and 30-day mortality tended to increase. Thus, healthcare centers with endoscopy services should consider the potential lethal clinical outcomes of patients requiring an endoscopic evaluation when adapting policy to cope with the ongoing COVID-19 pandemic.

## Figures and Tables

**Table 1:** Baseline characteristics of the overall population.

Characteristics	COVID-19 Pandemic (*n* = 60)	Pre-COVID-19 Pandemic (*n* = 460)	*p* Value	Standardized Difference
Male gender, *n* (%)	37 (61.7)	300 (65.2)	0.588	0.074
Age, mean (SD), year	67.0 (14.3)	62.8 (15.5)	0.045	0.285
Cirrhosis, *n* (%)	21 (35.0)	151 (32.8)	0.737	0.046
Chronic kidney disease, *n* (%)	17 (28.3)	95 (20.7)	0.174	0.179
Cardiovascular disease, *n* (%)	15 (25.0)	83 (18.0)	0.195	0.170
Cerebrovascular disease, *n* (%)	6 (10.0)	35 (7.6)	0.518	0.085
Malignancy, *n* (%)	18 (30.0)	83 (18.0)	0.028	0.283
Charlson comorbidity index, mean (SD)	5.1 (3.0)	4.4 (2.7)	0.039	0.273
Presenting symptom, *n* (%)				
Hematemesis	19 (31.7)	145 (31.5)	0.982	0.003
Coffee-ground emesis	20 (33.3)	166 (36.1)	0.676	0.058
Melena	38 (63.3)	300 (65.2)	0.774	0.039
Hematochezia	1 (1.7)	11 (2.4)	1.000	0.051
Maroon stool	2 (3.3)	24 (5.2)	0.756	0.093
Hemodynamic instability, *n* (%)	9 (15.0)	83 (18.0)	0.561	0.082
Medication, *n* (%)				
NSAIDs	6 (10.0)	115 (25.0)		0.403
Aspirin	12 (20.0)	107 (23.3)	0.010	0.079
Warfarin	6 (10.0)	49 (10.7)	0.572	0.021
Direct oral anticoagulant	0 (0)	1 (0.2)	0.877	0.069
Laboratory values on admission, median (IQR)				
Hemoglobin, g/dL				
Platelet, 10^3^/μL	7.5 (6.0–9.8)	8.1 (6.3–10.0)	0.320	−0.106
INR	160 (107–243)	198 (139–271)	0.007	−0.331
BUN, mg/dL	1.37 (1.25–3.03)	1.29 (1.09–2.74)	0.262	−0.045
Creatinine, mg/dL	38.1 (26.3–58.4)	32.1 (20.3–48.3)	0.059	0.233
Albumin, g/dL	1.09 (0.82–2.00)	1.06 (0.78–1.48)	0.330	0.146
Laboratory values on admission, median (IQR)	3.00 (2.58–3.50)	3.20 (2.80–3.70)	0.047	−0.295
Glasgow–Blatchford score, mean (SD)	10.8 (3.9)	10.7 (4.0)	0.834	0.041

BUN, blood urea nitrogen; INR, international normalized ratio; IQR, interquartile range; NSAIDs, nonsteroidal anti-inflammatory drugs; SD, standard deviation.

**Table 2:** Baseline characteristics of the matched population.

Characteristics	COVID-19 Pandemic (*n* = 46)	Pre-COVID-19 Pandemic (*n* = 138)	*p* Value	Standardized Difference
Male gender, *n* (%)	29 (63.0)	89 (64.5)	0.859	0.030
Age, mean (SD), year	65.7 (14.9)	63.3 (14.7)	0.337	0.163
Cirrhosis, *n* (%)	18 (39.1)	56 (40.6)	0.862	0.029
Chronic kidney failure, *n* (%)	9 (19.6)	28 (20.3)	0.915	0.018
Cardiovascular disease, *n* (%)	9 (19.6)	22 (15.9)	0.570	0.095
Cerebrovascular disease, *n* (%)	4 (8.7)	12 (8.7)	1.000	0.001
Malignancy, *n* (%)	12 (26.1)	32 (23.2)	0.690	0.067
Charlson comorbidity index, mean (SD)	4.8 (2.8)	4.6 (2.5)	0.614	0.084
Presenting symptom, *n* (%)				
Hematemesis	18 (39.1)	41 (29.7)	0.236	0.199
Coffee-ground emesis	13 (28.3)	54 (39.1)	0.185	0.232
Melena	31 (67.4)	89 (64.5)	0.721	0.061
Hematochezia	1 (2.2)	0 (0.0)	0.250	0.211
Maroon stool	1 (2.2)	9 (6.5)	0.455	0.214
Hemodynamic instability, *n* (%)	8 (17.4)	35 (25.4)	0.269	0.195
Medication, *n* (%)				
NSAID	6 (13.0)	17 (12.3)	0.898	0.022
Aspirin	8 (17.4)	35 (25.4)	0.269	0.195
Warfarin	4 (8.7)	14 (10.1)	1.000	0.050
Direct oral anticoagulant	4 (8.7)	17 (12.3)	0.503	0.118
Laboratory values on admission, median (IQR)				
Hemoglobin, g/dL	7.7 (6.0–10.0)	8.3 (6.3–10.3)	0.565	−0.041
Platelet, 10^3^/μL	167 (107–237)	181 (120–263)	0.421	−0.100
INR	1.4 (1.3–2.5)	1.3 (1.1–3.9)	0.664	−0.323
BUN, mg/dL	34.5 (18.4–53.7)	31.2 (2.2–49.2)	0.532	0.103
Creatinine, mg/dL	1.0 (0.8–1.4)	1.1 (0.8–1.7)	0.400	−0.083
Albumin, g/dL	3.1 (2.7–3.5)	3.1 (2.6–3.6)	0.893	0.032
Glasgow–Blatchford score, mean (SD)	10.5 (4.2)	10.8 (3.9)	0.700	−0.040

BUN, blood urea nitrogen; INR, international normalized ratio; IQR, interquartile range; NSAIDs, nonsteroidal anti-inflammatory drugs; SD, standard deviation.

**Table 3:** Causes of death among the overall population before and during the COVID-19 pandemic.

Cause of Death	COVID-19 Pandemic (*n* = 60)	Pre-COVID-19 Pandemic (*n* = 460)
Bleeding-related death	1 (1.7%)	12 (2.6%)
Nonbleeding-related death	10 (16.7%)	29 (6.3%)
Cardiovascular disease	1 (1.7%)	2 (0.4%)
Infection	3 (5.0%)	14 (3.0%)
Extra-gastrointestinal malignancy	6 (10.0%)	13 (2.8%)

Note. Data are presented as the number (percentage) of a condition.

**Table 4:** Treatment outcomes for the overall and matched populations.

Variable	Overall Population						Matched Population					
	COVID-19 Pandemic (*n* = 60)	Pre-COVID-19 Pandemic (*n* = 460)	Unadjusted OR/Difference (95% CI) *	*p* Value *	Adjusted OR/Difference (95% CI) †	*p* Value †	COVID-19 Pandemic (*n* = 46)	Pre-COVID-19 Pandemic (*n* = 138)	Unadjusted OR/Difference (95% CI) *	*p* Value *	Adjusted OR/Difference (95% CI) ^ψ^	*p* Value ^ψ^
30-day mortality, *n* (%)	11 (18.3)	41 (8.9)	2.29 (1.11–4.75)	0.025	1.33 (0.52–3.39)	0.550	8 (17.4)	16 (11.6)	1.61 (0.64–4.04)	0.315	1.92 (0.71–5.19)	0.199
Endoscopy, *n* (%)	26 (43.3)	439 (95.4)	0.04 (0.02–0.07)	<0.001	0.01 (0.003–0.03)	<0.001	19 (41.3)	135 (97.8)	0.02 (0.004–0.06)	<0.001	0.02 (0.003–0.06)	<0.001
Endoscopy within 24 h, *n* (%)	4 (6.7)	208 (45.2)	0.09 (0.03–0.24)	<0.001	0.06 (0.02–0.22)	<0.001	3 (6.5)	68 (49.3)	0.07 (0.02–0.24)	<0.001	0.07 (0.02–0.24)	<0.001
Rebleeding, *n* (%)	8 (13.3)	41 (8.9)	1.68 (0.74–3.79)	0.212	1.16 (0.44–3.03)	0.764	6 (14.0)	16 (11.9)	1.21 (0.44–3.31)	0.716	1.34 (0.44–4.03)	0.605
Blood transfusion, median (IQR), unit	3 (1–6)	2 (1–3)	2.38 (1.78–2.99)	<0.001	1.18 (0.60–1.75)	0.051	3 (1–5)	2 (1–3)	1.04 (0.51–1.56)	0.153	1.08 (0.54–1.63)	0.144
Length of stay, median (IQR), day	5 (4–12)	2 (1–3)	4.17 (2.14–6.19)	<0.001	2.21 (0.11–4.31)	0.198	5 (3–9)	4 (2–6)	2.0 (1.02–2.98)	0.045	2.17 (1.13–3.20)	<0.001

Abbreviations: OR, odds ratio; CI, confidence interval; IQR, interquartile range. * The *p* values for mortality, endoscopy, and rebleeding rates were determined using logistic regression analysis. The *p* values for blood transfusion and length of stay were determined using the linear regression analysis. † Adjusted for variables with standard difference >0.15 among the overall population, including age, chronic kidney disease, cardiovascular disease, malignancies, Charlson comorbidity index, use of nonsteroidal anti-inflammatory drugs, serum levels of albumin, blood urea nitrogen, and platelet count. **^ψ^** Adjusted for variables with standard difference >0.15 among the matched population, including age, hematemesis, coffee-ground emesis, hematochezia, maroon stool, hemodynamic instability, use of aspirin, and international normalized ratio.

**Table 5:** The need for endoscopy and blood transfusion among the overall population according to the Glasgow–Blatchford score.

The Glasgow–Blatchford Score	COVID-19 Pandemic (*n* = 60)			Pre-COVID-19 Pandemic (*n* = 460)		
	No. of Patients	In-Hospital Endoscopy	Blood Transfusion	No. of Patients	In-Hospital Endoscopy	Blood Transfusion
0	1	0	0	7	6	1
1	2	0	0	8	6	1
2	0	0	0	12	8	2
3	0	0	0	11	10	3
4	3	1	1	3	3	1
5	2	1	1	6	5	1
6	0	0	0	17	16	7
7	3	1	3	30	27	23
8	2	2	1	31	29	19
9	3	2	2	23	22	19
≥10	44	19	41	312	307	283

**Table 6:** Endoscopic findings among the overall and matched populations.

Endoscopic Finding	Overall Population				Matched Population			
	COVID-19 Pandemic(*n* = 26) *	Pre-COVID-19 Pandemic(*n* = 439) *	*p* Value	Standardized Difference	COVID-19 Pandemic(*n* = 19) *	Pre-COVID-19 Pandemic(*n* = 135) *	*p* Value	Standardized Difference
Peptic ulcer disease, *n* (%)	10 (38.5)	240 (54.7)	0.107	0.329	8 (42.1)	72 (53.3)	0.359	0.226
Active bleeding	1 (3.8)	19 (4.3)			1 (5.3)	3 (2.2)		
Nonbleeding visible vessel	0 (0.0)	31 (7.1)			0 (0.0)	12 (8.9)		
Clot with underlying vessel	0 (0.0)	19 (4.3)			0 (0.0)	9 (6.7)		
Pigmented spot/clean base	9 (34.6)	199 (45.3)			7 (36.8)	57 (42.2)		
Varices, *n* (%)	10 (38.5)	128 (29.2)	0.313	0.198	7 (36.8)	48 (35.6)	0.913	0.028
Others, *n* (%)	7 (26.9)	98 (22.3)	0.586	0.107	5 (26.3)	25 (18.5)	0.535	0.188

* Some patients presented with more than one endoscopic finding.

## Data Availability

All the data are included in the manuscript.
